# High abundance of BDNF within glutamatergic presynapses of cultured hippocampal neurons

**DOI:** 10.3389/fncel.2014.00107

**Published:** 2014-04-11

**Authors:** Thomas Andreska, Sarah Aufmkolk, Markus Sauer, Robert Blum

**Affiliations:** ^1^Institute for Clinical Neurobiology, University Hospital, Julius-Maximilians-University WürzburgWürzburg, Germany; ^2^Department of Biotechnology and Biophysics, Biocenter, Julius-Maximilians-University WürzburgWürzburg, Germany

**Keywords:** BDNF, synaptic localization, presynapse, hippocampal neurons, synapse structure

## Abstract

In the mammalian brain, the neurotrophin brain-derived neurotrophic factor (BDNF) has emerged as a key factor for synaptic refinement, plasticity and learning. Although BDNF-induced signaling cascades are well known, the spatial aspects of the synaptic BDNF localization remained unclear. Recent data provide strong evidence for an exclusive presynaptic location and anterograde secretion of endogenous BDNF at synapses of the hippocampal circuit. In contrast, various studies using BDNF overexpression in cultured hippocampal neurons support the idea that postsynaptic elements and other dendritic structures are the preferential sites of BDNF localization and release. In this study we used rigorously tested anti-BDNF antibodies and achieved a dense labeling of endogenous BDNF close to synapses. Confocal microscopy showed natural BDNF close to many, but not all glutamatergic synapses, while neither GABAergic synapses nor postsynaptic structures carried a typical synaptic BDNF label. To visualize the BDNF distribution within the fine structure of synapses, we implemented super resolution fluorescence imaging by direct stochastic optical reconstruction microscopy (*d*STORM). Two-color *d*STORM images of neurites were acquired with a spatial resolution of ~20 nm. At this resolution, the synaptic scaffold proteins Bassoon and Homer exhibit hallmarks of mature synapses and form juxtaposed bars, separated by a synaptic cleft. BDNF imaging signals form granule-like clusters with a mean size of ~60 nm and are preferentially found within the fine structure of the glutamatergic presynapse. Individual glutamatergic presynapses carried up to 90% of the synaptic BDNF immunoreactivity, and only a minor fraction of BDNF molecules was found close to the postsynaptic bars. Our data proof that hippocampal neurons are able to enrich and store high amounts of BDNF in small granules within the mature glutamatergic presynapse, at a principle site of synaptic plasticity.

## Introduction

Brain-derived neurotrophic factor (BDNF), the predominant member of the neurotrophin family in the adult brain, has a strong impact on neuronal differentiation and plasticity (Barde et al., 1982; Soppet et al., 1991; Liu et al., 1995; Thoenen, 1995; Luikart and Parada, 2006; Park and Poo, 2012). In the brain, BDNF is not a major survival factor, but mediates region-specific effects on synaptic function and neuronal complexity (Nikoletopoulou et al., 2010; Rauskolb et al., 2010). For instance, loss of striatal BDNF signaling causes defects in the development of striatal medium spiny neurons accompanied by reduced dendritic complexity, and a reduced spine density (Rauskolb et al., 2010; Li et al., 2012). In contrast, in the hippocampus, a brain region with comparatively high BDNF mRNA and protein levels (Conner et al., 1997), only minor changes in neuronal anatomy were found when BDNF was removed either from all cells (Korte et al., 1995) or all neurons (Rauskolb et al., 2010).

In the hippocampus and the emotional circuit, BDNF/TrkB signaling is involved in the induction of long-term potentiation (LTP), a prototypical model to study synaptic plasticity, cellular learning, and memory (Korte et al., 1995; Figurov et al., 1996; Kossel et al., 2001; Kovalchuk et al., 2002; Zakharenko et al., 2003; Nicoll and Schmitz, 2005; Huang et al., 2008; Meis et al., 2012). Indeed, in humans a specific polymorphism in the *bdnf* gene, the Val66Met polymorphism, reduces levels of activity-dependent BDNF secretion and affects episodic memory, hippocampal function (Egan et al., 2003), and learning in the emotional circuit (Chen et al., 2006; Lonsdorf and Kalisch, 2011).

While BDNF effects at synapses are beginning to be well defined (Blum and Konnerth, 2005; Luikart and Parada, 2006; Park and Poo, 2012), attempts to localize BDNF were complicated by the low amounts of endogenous BDNF normally found in neurons *in vivo* (Dieni et al., 2012; Park and Poo, 2012). Thus, neuronal cultures became a routine tool to study activity-dependent BDNF synthesis, synaptic steady-state localization and BDNF-release mechanisms (Zafra et al., 1990; Blöchl and Thoenen, 1996; Canossa et al., 2001; Hartmann et al., 2001; Kohara et al., 2001; Balkowiec and Katz, 2002; Brigadski et al., 2005; Matsumoto et al., 2008; Lessmann and Brigadski, 2009; Matsuda et al., 2009; Yang et al., 2009; Park and Poo, 2012). However, BDNF levels are low in neuronal cultures as well, thus vector-driven expression of recombinant BDNF became the method of choice to support BDNF detection (Canossa et al., 2001; Hartmann et al., 2001; Kohara et al., 2001; Gärtner and Staiger, 2002; Chen et al., 2004; Adachi et al., 2005; Brigadski et al., 2005; Santi et al., 2006; Dean et al., 2009; Matsuda et al., 2009; Cheng et al., 2011; Petoukhov et al., 2013). Most of these studies came to the conclusion that BDNF is stored and released from both axonal and dendritic compartments. Data obtained with GFP-tagged versions of BDNF suggested that postsynaptic secretory granules close to glutamatergic synapses are a preferential site of BDNF release (Hartmann et al., 2001; Brigadski et al., 2005; Lessmann and Brigadski, 2009). This is supported by the finding that BDNF transcripts were found in dendrites of hippocampal neurons *in vitro* and *in vivo* as well (Tongiorgi et al., 1997; An et al., 2008; Chiaruttini et al., 2009). In contrast, previous and recent work visualized endogenous BDNF at presynaptic sites in brain sections, corroborating synaptic effects of BDNF by anterograde release (Altar et al., 1997; Conner et al., 1997; Danzer and McNamara, 2004; Dieni et al., 2012).

In order to clarify whether BDNF is preferentially available in the presynapse, the postsynapse or at extrasynaptic sites, we investigated the synaptic localization of endogenous BDNF in long-term cultured hippocampal neurons (>21–38 days *in vitro*). Confocal fluorescence microscopy revealed BDNF labeling close to glutamatergic synapses, however this method failed to provide the optical resolution to answer the question whether BDNF resides close to, or within the fine structure of synapses. Therefore, we used *direct* STORM (*d*STORM), a super-resolution fluorescence imaging approach enabling spatial lateral resolutions of ~20 nm, (Heilemann et al., 2008; Van De Linde et al., 2011).

Two-color *d*STORM localized the vast majority of endogenous synaptic BDNF molecules in small granules within presynaptic glutamatergic terminals. Thus, our results corroborate the finding that high amounts of synaptic BDNF are available for a regulated anterograde release from glutamatergic synapses.

## Materials and methods

### Animals

All experimental procedures were approved by our institutional animal care and utilization committee and done in accordance with guidelines by the European Union. Hippocampal cultures were established from CD-1, with the exception of cultures from BDNF^fl/fl^ mice (Rauskolb et al., 2010). These mice were kept on a C57BL/6J background. All mice were maintained under a 12 h light/dark cycle with food and water *ad libitum*.

### Primary hippocampal neurons

Hippocampi of mice at postnatal day 1–2 were collected in Hank's buffered saline solution (Sigma) and trypsinized with 1% trypsin (Worthington; 50 μl per hippocampus) in PBS at 37°C for 15 min. Trypsinization was stopped with 50 μl 1% trypsin inhibitor (Sigma) in PBS. Cells were dissociated mechanically using fire-polished Pasteur pipettes. Cells were pelleted for 3 min at 400× g, washed three times in Neurobasal medium (Invitrogen), and finally resuspended in Neurobasal medium with 2x B27 supplement (Invitrogen) and 1x GlutaMAX (Invitrogen). In a later stage of the study, we used 1x B27 supplement (Invitrogen) and 1x N2 supplement (Invitrogen). 10,000–25,000 cells were grown on 10 mm glass coverslips (Marienfeld) or glass-bottom imaging chambers (Nunc), coated with 0.1% poly-L-lysine (Sigma). Coated glass coverslips or imaging chambers were never dried and stored in Hanks buffered saline solution at 4°C. Half of the growth medium was replaced once per week. Lentiviral transduction of neurons harvested from *bdnf*^*fl/fl*^ mice was performed at day one *in vitro* (DIV 1).

### Lentiviral particles

Lentiviral vectors based on self-inactivating LV-(CMV-promoter driven Nls-Cre^IRES^TDtomato) or FU-backbones (ubiquitin-promoter driven luminal GFP). Lentiviral vectors were produced in HEK293T cells using the pseudotyping vector pMD2.G and pCMVDR8.91. Lentiviral particles were concentrated using ultracentrifugation and stored at -80°C in (in mM) 50 Tris-HCl, pH 7.8, 130 NaCl, 10 KCl, 5 MgCl_2_.

### Western blot analysis of BDNF

Hippocampi were lysed in extraction buffer (50 mM sodium acetate, 1M NaCl, 0,1% Triton X100, pH 4,0, protease inhibitor tablets), and by means of a Sonifier (UP50H Hielscher) with a MS1 sonotrode. Cell lysates were cleared at 20.000× g for 10 min at 4°C. Proteins were blotted on Immun-Blot PVDF membranes (Biorad). Blocking and antibody incubation were performed in 10% goat serum, 5% milk powder (Biorad) for 3–4 h. For BDNF detection, rabbit anti-BDNF 17H (1/6000, provided by Michael Sendtner, Institute for Clinical Neurobiology, Würzburg, Germany) was incubated over night at 4°C. Anti-Cytochrome C A-8 (Santa Cruz, 0.2 μg/ml) was used as loading control. For detection, the ECL Plus kit (GE Healthcare) was used in combination with horseradish peroxidase-coupled secondary antibodies (Jackson Laboratories).

### Indirect immunofluorescence labeling for confocal microscopy

Cells were washed with PBS and fixed with 4% PBS-buffered paraformaldehyde for 15 min at 37°C. Cells were washed with PBS and treated with blocking solution (1x PBS, 10% BSA, 0.1% Triton X100, 0.1% Tween 20 or 1x PBS, 10% horse serum, 0.1% Triton X100) and incubated at RT for at least 30 min. Primary antibodies were diluted in blocking solution to working concentration. Primary antibodies were incubated for 2–3 h at RT, then washed 8x with washing solution (1x PBS, 0.1% Triton X100, 0.1% Tween 20). Finally cells were washed and cell nuclei were labeled with DAPI (0.4 μg/ml). For confocal laser-scanning microscopy, cells were embedded in Aquapolymount solution (Polysciences).

Primary antibodies: *Mouse* anti-BDNF mab248 (RDsystems, 1/500); -BDNF GF35L (Oncogene, 1/500) -Map2 AP20 (Sigma, 0.5 μg/ml), -BDNF mab#9 (Kolbeck et al., 1999) (provided by Yves-Alain Barde, Biocenter, Basel Switzerland); -Bassoon mab7f (Tom Dieck et al., 1998) provided by Eckart Gundelfinger, Magdeburg, Germany, 1/2000); -Synapsin (Synaptic systems, 1/2000). *Rabbit* anti-proBDNF (Alomone, 1/1000), -BDNF 17h (provided by Michael Sendtner, Institute for Clinical Neurobiology, University of Würzburg, Germany; at 1/1000), -Bassoon sap7f (Bockmann et al., 2002) (Eckart Gundelfinger, Magdeburg, 1/2000); anti-Homer 1 (Synaptic System; at 1/400); anti ProSAP1 (Eckart Gundelfinger, Magdeburg, 1/1000); vGAT (Synaptic systems, 1/2000); -vGlut1 (BNPI) (Synaptic systems, 1/2000); *chicken* anti-Neurofilament (Millipore, 1/10000) at 1/1000; *sheep* anti-BDNF (Chemicon ab1513-1/500.); *chicken* anti-Map2 (Abcam, 1 μg/ml); *guinea pig* anti-vGAT (Synaptic systems, 1/400); -vGlut (Synaptic systems) at 1/2000. For indirect immunofluorescence we used the following secondary antibodies at a concentration of 0.7 μg/ml: Dylight 405, Dylight 488, Dylight 549, Cy3, Cy5, Dylight 649 (Jackson laboratories) or Alexa488, Alexa568, and Alexa647 (Invitrogen).

### Confocal laser scanning microscopy and image processing

Images were acquired using an inverted IX81 microscope equipped with an Olympus FV1000 confocal laser scanning system, a FVD10 SPD spectral detector and diode lasers of 405, 473, 559, and 635 nm. All images shown were acquired with an Olympus UPLSAPO60x (oil, numerical aperture: 1.35) objective. For high-resolution confocal scanning a pinhole setting representing one Airy disc was used. High-resolution confocal settings were chosen to meet an optimum resolution of at least 3 pixels per feature in x–y direction. In z-direction, 300 nm steps were used. 12-bit Z-stack images were processed by maximum intensity projection and were adjusted in brightness and contrast using Image J software (Rasband, W. S., ImageJ, U. S. National Institutes of Health, Bethesda, Maryland, USA, http://imagej.nih.gov/ij/, 1997–2012). In some cases, maximum intensity projection images were adjusted by a slight gamma-correction from 1.0 to 0.8 to improve the presentation. Images are shown as RGB images (8-bit per color channel).

### Colocalization analysis

Pixel intensity-based colocalization of antibody-derived fluorescent signals was performed with an unbiased algorithmic method in two-color three-dimensional confocal images. Data represent the mean Pearson's correlation coefficient (*r* ± *SD*) for pixels where both channels, antibody one in Ch1 and antibody two in Ch2, are above a calculated intensity threshold at which pixels do not show any statistical correlation (Costes et al., 2004).

### *d*STORM imaging

Details of the microscope setup are described (Van De Linde et al., 2011). Briefly, fluorescence imaging was performed on an inverted microscope (IX71; Olympus) equipped with an oil-immersion objective (60x, NA 1.45; Olympus). For excitation we used a 641 nm (Cube 640-100C; Coherent) and a 532 nm diode laser (Nano250; Linos), applying irradiation intensities of 1–5 kW/cm^2^. The laser beams were overlaid by dichroic mirrors (LaserMUX 514-543, LaserMUX 633-647; Semrock). For spectral separation of fluorescence and excitation light a dichroic beam-splitter (HC 560/659; Semrock) was used. The fluorescence of Alexa647 and Alexa532 was split by a dichroic mirror (DCXR630, Chroma) at 630 nm and spectrally filtered using suitable bandpass filters (HQ582/75; Semrock) for Alexa532, (HC697/75 and a longpass filter LP647RS; Semrock) for Alexa647 positioned in front of a shorter and longer wavelength electron-multiplying charge-coupled-device (EMCCD) camera (Ixon DU897; Andor), respectively. Before imaging the storage medium was exchanged by PBS-based photoswitching buffer containing 100 mM β-mercaptoethylamine (MEA) adjusted to pH 8.0–8.3 (Van De Linde et al., 2011). Experiments were performed by internal reflection fluorescence (TIRF)-microscopy. The experimental spatial resolution of ~20 nm was determined from individual signals of the above mentioned fluorophore labeled antibodies [see also Van De Linde et al., 2011].

### *d*STORM image analysis

*d*STORM images were reconstructed with the open-source software rapidSTORM 2.21 (Wolter et al., 2010, 2012). Only fluorescence signals with more than 500 detected photons per frame (Alexa532 and Alexa647) were analyzed. By analyzing their ellipticity, multi-fluorophore events were excluded from further analysis. To achieve a higher image precision we pooled fluorophore signals that stay in more than one sequential frame in the fluorescent state into one localization with a cumulative photon number. This prevented an adulterated number of localizations. The *d*STORM images were reconstructed with a pixel size of 10 nm. The color intensity is directly proportional to the localization density per pixel. Two-color *d*STORM images were aligned by a non-linear transformation matrix via bUnwarbJ (Arganda-Carreras et al., 2006). Calibration measurements to create a transformation matrix were performed with 200 nm fluorescent microspheres (TetraSpeck; Life Technologies). Final images were adjusted in brightness and contrast using Image J software (Rasband, W. S., ImageJ, U. S. National Institutes of Health, Bethesda, Maryland, USA, http://imagej.nih.gov/ij/, 1997–2012).

### Two-color *d*STORM image analysis

We investigated the overlap of BDNF with vGlut as presynaptic and Homer as postsynaptic marker. Two localizations were considered as overlapping when the distance between the localizations in the x,y-direction was less than 20 nm, approximately corresponding to the spatial resolution achieved in our experiments. Next, we created a binary mask for the two color-channels. We applied a Gaussian blur with a sigma of 10 nm and a threshold with a pixel value of 0.25 to account for the fact that the rapidSTORM algorithm divides a localization that is not exactly in the middle of a pixel prorated on four pixels. The extreme event occurs when the localization is exactly located in between four pixels. Therefor the localization gets divided in equal shares on all four pixels which results in a pixel value of 0.25. After the transformation of intensity values into binary information (Figure 7B) we determined the percentage of overlapping areas for BDNF with the binary masks for pre- and postsynaptic markers (Figure 7C).

### Determination of the size and number of BDNF clusters

The x,y-dimension of the BDNF IR was determined in the binary image masks (Figure 7B). Next, we adapted the BDNF mask on a grayscale image in which the gray value is equal to the exact number of localizations per pixel. We defined a BDNF+ cluster as a BDNF+ element of 2 or more localizations. As the BDNF localizations formed almost spherical image elements, we determined the diameter of BDNF IR clusters using the following equation: $2*\sqrt{Area/\pi }$. We finally determined the mean value and median of the diameter of all analyzed BDNF+ clusters (see Figures 7E,F).

### Image presentation

For presentation, figures were prepared with Adobe Photoshop CS5.

## Results

### Antibody selection

**Figure 1:**
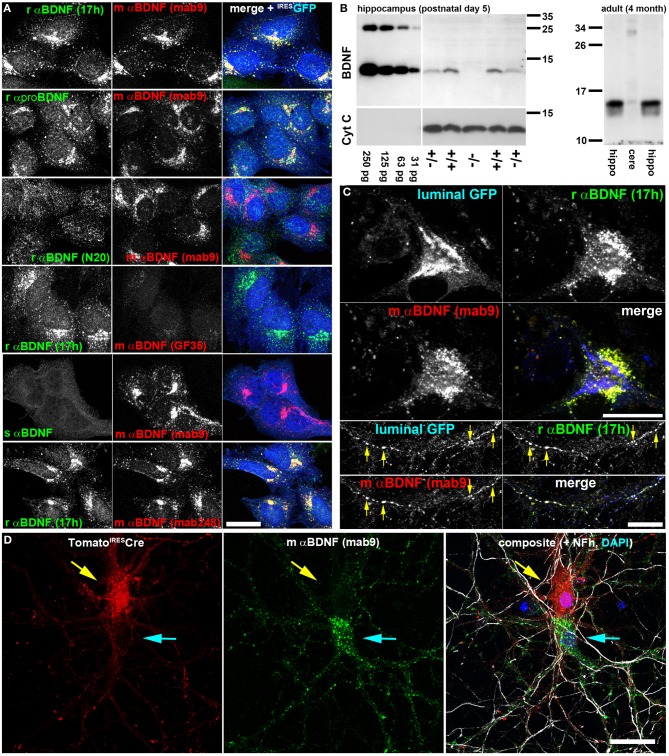
**Two anti-BDNF antibodies enable specific and dense indirect immunolabeling of endogenous BDNF in hippocampal neurons *in vitro*. (A)** Antibody verification with recombinant BDNF. HeLa cells stably expressing lentivirally-delivered BDNF^IRES^GFP were labeled with different anti (α)-BDNF antibodies from different species (r, rabbit; m, mouse; s, sheep). Antibody details are given in the Material and Method section. As transduction control, GFP was expressed bicistronically under an IRES2 sequence and postlabeled with chicken anti-GFP. BDNF was accumulated in the early secretory trafficking pathway after microtubule disruption with nocodazole, causing a perinuclear Golgi-like staining pattern. **(B)** Immunoblot of rαBDNF (17 h). Serial dilutions of recombinant BDNF (250–31 pg per lane) served as control (left panel). Hippocampal BDNF harvested at postnatal day 5 was absent in *bdnf*^*−/−*^ mice, while heterozygous and wildtype controls show mature BDNF at a M_*r*_ of 13 kDa. HRP-conjugated anti-Cytochrome C (Cyt C) served as loading control (left panel). In adult mice, rαBDNF (17 h) revealed mature BDNF at high concentration in the hippocampus (left and right lane), while only low amounts were detected in cerebellar protein lysates (middle lane). (right panel). Immunoblots revealed high specificity of the antibody 17 h. **(C)** Endogenous BDNF in hippocampal neurons at day in vitro (DIV) 35. In somatic regions, BDNF was not concentrated in perinuclear Golgi cisternae, but rather in somatic vesicles. Golgi cisternae were labeled with luminal GFP, a marker for the anterograde, secretory pathway. Luminal GFP and endogenous BDNF co-labeled anterograde trafficking structures (arrow heads) in neurites of hippocampal neurons. Endogenous BDNF was double-labeled by two different anti-BDNF antibodies (rabbit anti-BDNF 17h and mouse anti-BDNF mab9). **(D)** Somatic anti-BDNF labels were lost when the *bdnf* gene was removed from single cells. Hippocampal neurons from bdnf^fl/fl^ mice were transduced with a low titer of a lentivirus expressing tdTomato^IRES^Cre. Neurons (DIV 20) were labeled with anti-BDNF (mab9). Tomato+ cells (upper neuron, red, yellow arrow) lacked a typical vesicular somatic BDNF label, while untransduced, Tomato-Cre-deficient neurons (lower neuron, cyan arrow) exhibited a strong somatic BDNF label. In the composite image Tomato (red) and mouse anti-BDNF (mab9) (green) labels are shown together with an anti-neurofilament (Nfh) label (white) and a nuclear DAPI stain (blue). Bar: 25 μm.

Initially we screened different anti-BDNF antibodies to ensure a dense immunoreactivity (IR) of BDNF. We transduced HeLa cells with a lentiviral vector expressing BDNF. To identify transduced cells, cytosolic GFP was bi-cistronically expressed under control of an internal ribosomal entry site. 72 h after transduction, cells were treated with nocodazole to block the constitutive anterograde BDNF release through the secretory pathway (Blum et al., 2000). Cells were labeled with two different anti-BDNF antibodies per specimen and cells expressing BDNF^IRES^GFP were identified by the cytosolic GFP label. Confocal analysis identified three antibodies providing a dense BDNF IR in the secretory pathway: rabbit anti-BDNF (17h), -(mab#9) (Kolbeck et al., 1999; Dieni et al., 2012), and -Mab248 (RD systems) (Figure 1A). To verify colocalization between two antibody-derived labels, we determined the Pearson's correlation coefficient (*r* ± *SD*) after an unbiased estimation of a threshold value for identifying background levels in each color channel (Costes et al., 2004; Bolte and Cordelieres, 2006). A Pearson's correlation coefficient (*r*) of 1.0 stands for 100% colocalization while *r* = 0 represents a random overlap of pixel intensities. Regions-of-interest (ROIs), representing individual cells, were determined by the cytosolic GFP label. Mouse anti-BDNF-Mab#9 showed a high amount of colocalization with anti-rabbit-BDNF-17 h (*r* = 0.83 ± 0.05; *n* = 11), and with rabbit anti-proBDNF (*r* = 0.79 ± 0.05; *n* = 11). Anti-rabbit BDNF (N20) failed to colocalize with mouse-anti-BDNF (Mab#9) (*r* = 0.02 ± 0.09; *n* = 16). Anti-BDNF (Mab248) and 17h showed an excellent overlap as well (*r* = 0.81 ± 0.03; *n* = 9), however, anti-BDNF mab248 failed to provide anti-BDNF IR in mouse hippocampal neurons or hippocampal slices, and was therefore excluded from further investigations.

Next we tested the specificity of anti-BDNF 17 h by Western blot analysis of BDNF knockout mice (Korte et al., 1995). Acidic protein lysates of hippocampal tissue of *bdnf ^ko/ko^*-, *bdnf ^wt/ko^* mice and wild type littermates were probed with anti-BDNF 17 h (*n* = 3). Serial dilutions of recombinant BDNF and Cytochrome C served as Western blot controls. Protein lysates from heterozygous *bdnf ^wt/ko^* mice and wild type control showed low amounts of BDNF in the expected size of ~13 kDa, while *bdnf ^ko/ko^* mice showed no BDNF Western signal (Figure 1B). At this early postnatal stage (postnatal day 5, P 5), when *bdnf ^ko/ko^* mice were still viable, we obtained approximately 10–20 pg of BDNF per 40 μg of acidic protein lysate from wild type littermates. BDNF expression starts to increase postnatally and becomes high at approximately three weeks after birth (Maisonpierre et al., 1990). In protein lysates of 4-month-old C57B/6 mice, a strong and specific BNDF signal was observed in the hippocampus, while only low amounts of BDNF protein were found in the cerebellum.

To highlight the anterograde secretory pathway, we used luminal GFP as a trafficking marker (Blum et al., 2000). After release from the ER-to-Golgi intermediate compartment, luminal GFP concentrates in the subsequent anterograde secretory pathway (Blum et al., 2000). In neuronal somata both BDNF labels (17 h vs. mab#9) co-localized, and BDNF was found in perinuclear vesicular membranous structures (Figure 1C). Luminal GFP marked the typical cisternae of the perinuclear Golgi apparatus. Luminal GFP did not co-localize with somatic BDNF, while all three labels, luminal GFP, anti-BDNF 17 h and anti-BDNF mab#9 IR, stained the same secretory transport containers in cultured neurons (Figure 1C, yellow arrows).

**Figure 2:**
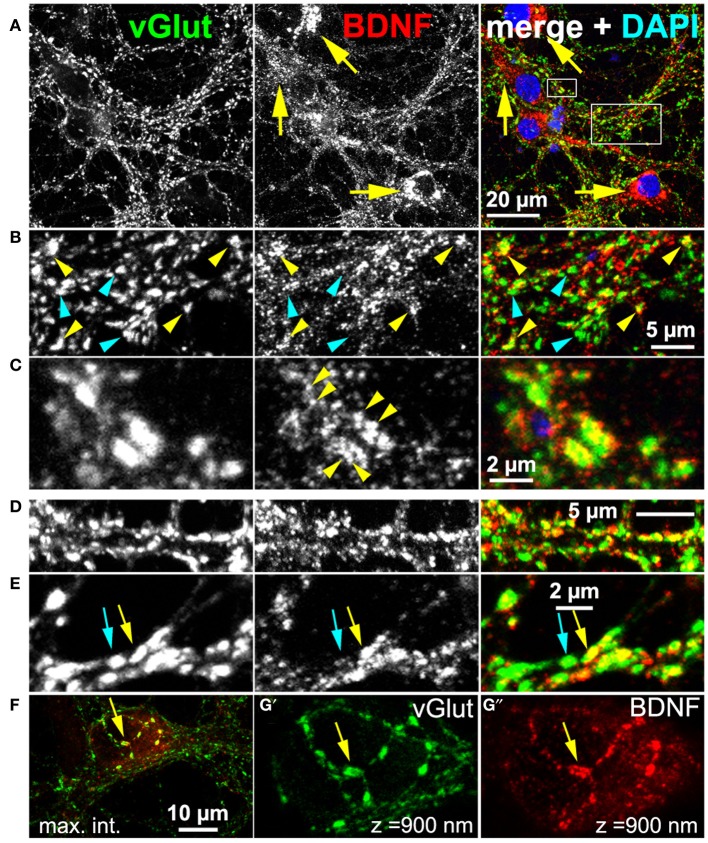
**Endogenous BDNF at glutamatergic synapses (DIV 38). (A–C)** Hippocampal neurons with high amounts of BDNF in somatic regions (yellow arrows). **(B) (**detail of **A)**, BDNF IR was found in close proximity to some (yellow arrowheads), but not all vGlut+ synapses (cyan arrowheads). **(C) (**detail of **A)**, Single vGlut+ structures exhibited a dense, polymorphic, vesicle-like BDNF signal (yellow arrowheads in middle panel). **(D,E)** vGlut+/BDNF-rich (yellow arrow) and vGlut+/BDNF-poor synapses (cyan arrow) co-existed in close proximity. **(F–G)** Neuronal soma with many vGlut+/BDNF-poor synapses in the somatic periphery, innervated by a projection with multiple vGlut+/BDNF-rich synapses (yellow arrow). In **(G')** and **(G”) (**detail of **F)**, a confocal stack of 900 nm (maximum intensity projection) from the surface of the somatic region is shown. In **(A–C)**, cell nuclei were labeled with DAPI and are shown in blue.

**Figure 3:**
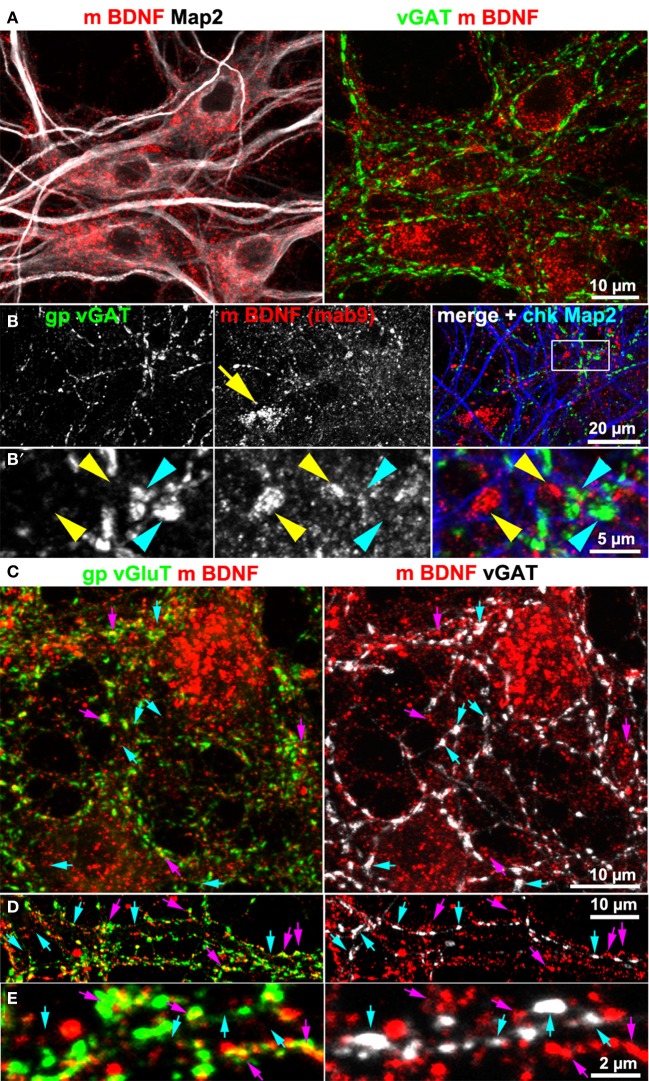
**BDNF immunoreactivity is low at GABAergic synapses. (A,B)** vGAT and Map2 staining of hippocampal neurons (DIV 25–DIV 31). vGAT+ structures did not show BDNF enrichment. Somatic BDNF staining **(**yellow arrow in **B)** was not associated with vGAT+ neurons. **(B') (**detail in **B)**. Dense anti-BDNF staining (yellow arrowheads) was almost absent from vGAT+ structures (cyan arrowheads). **(C–E)** In contrast to vGlut-positive structures, vGAT+ neurites barely overlapped with BDNF IR. Cyan arrows point to vGAT+/BDNF-poor synapses. Purple arrowheads point to a close proximity between vGlut and BDNF. Pearson's correlation coefficients describing the overlap between the immune labels are given in the text.

To enable cell-specific ablation of BDNF expression by Cre-mediated ablation of the floxed *bdnf* gene from *bdnf*^*fl/fl*^ mice (Rauskolb et al., 2010), we transduced corresponding neurons with a low titer of a bicistronic lentiviral construct expressing both the red fluorescent reporter protein tdTomato and nuclear-targeted Cre recombinase (tdTomato^IRES^Cre). Transduced neurons no longer showed the typical vesicular-membranous BDNF IR in the soma (Figure 1D, yellow arrow). Non-transduced neurons in the vicinity (cyan arrow in Figure 1D) showed typical somatic BDNF labeling (Figures 1–3). These patterns reveal that somatic BDNF detected by anti-BDNF 17h and mab#9 represents the cell-autonomous protein expression of BDNF. More importantly, these data show the high specificity of the anti-BDNF antibody mab#9 (Kolbeck et al., 1999) for our study.

The following antibodies failed to fulfill our criteria for a dense and specific labeling of endogenous mouse BDNF: N20 (Santa Cruz); GF35 (Oncogene), sheep anti-BDNF (Chemicon) (Figure 1A), as well as rabbit anti-BDNF H-117 (Santa Cruz), and chicken anti BDNF (RD Systems).

### BDNF at synaptic sites of hippocampal neurons

BDNF function implicates that BDNF is present at glutamatergic synapses. For this reason, we performed a confocal analysis of cultured hippocampal neurons labeled with anti-BDNF (mab#9) and anti-vGlut1 (vesicular glutamate transporter) (Figure 2). The latter antibody served as presynaptic marker for glutamatergic synapses. We used long-term cultured neurons (here 5 weeks), which tend to have pronounced attributes of synapse maturity (Orefice et al., 2013). These neurons are able to establish pre- and postsynaptic bar structures (see this study) as a typical sign of synapse maturity (Dani et al., 2010). Neurons exhibited a pronounced somatic, vesicular-membranous BDNF staining (Figure 2A, yellow arrows) and no perinuclear vGlut signal. To determine the number of neurons exhibiting this typical somatic perinuclear BDNF staining, we labeled neurons with Map2 and BDNF and imaged areas of ~320 μm^2^ with confocal microscopy at a resolution of 320 nm/pixel (35 areas, 376 neurons, n = 4). We found an average of 11 Map2+ neurons per area and 92% of all Map2+ neurons showed the typical pronounced perinuclear BDNF IR. In the periphery, multiple vGlut+ synapses showed only minor BDNF IR (Figure 2B, cyan arrowheads), but in several vGlut+ synapses a substantial amount of BDNF was found in close proximity to vGlut (Figure 2B, yellow arrowheads). While vGlut was homogenously distributed in theses synaptic areas, BDNF IR appeared in densely packed, immunoreactive clusters (Figure 2C, yellow arrowheads). This reveals that BDNF IR can be found in close proximity to vGlut+ synapses, and overlaps with vGlut IR, but BDNF IR does not directly colocalize with the vGlut signal (Figure 2C, yellow arrowheads). Interestingly, vGlut+/BDNF-rich (yellow arrow) and vGlut+/BDNF-poor synapses (cyan arrow) co-existed in close vicinity (Figure 2E). vGlut+/BDNF-poor synapses are rare in the periphery of neuronal somata (Figure 2F). A frequent observation were typical axonal projections with vGlut+/BDNF-rich synapses at the upper surface of the hippocampal soma (Figures 2G',G”, confocal z-stack of 900 nm, yellow arrow).

Hippocampal cultures also contain GABAergic neurons. For this reason we tested BDNF IR at GABAergic synapses. We never found accumulation of BDNF IR in synapses positive for the vesicular GABA transporter (vGAT; Figure 3). Neither vGAT+ synapses close to BDNF+ somata (Figure 3A), nor vGAT-positive structures in peripheral neurites showed a substantial BDNF label (Figures 3B,B'). Next we stained vGlut, vGAT and BDNF, acquired high-resolution confocal z-stacks (e.g., Figures 3C–E) and calculated whether BDNF labels correlate with either vGlut+ or vGAT+ structures. After intensity-threshold determination (Costes et al., 2004), anti-BDNF and anti-vGlut labels showed a non-random correlation (Pearson's correlation coefficient *r* = 0.24 ± 0.04 (*SD*, *n* = 9) of overlapping structures. In contrast, no correlation was seen between anti-BDNF and anti-vGAT labels (*r* = 0.01 ± 0.01 *SD*, *n* = 9). This value was equal to the correlation coefficient comparing vGAT+ and vGlut+ labels (*r* = 0.01 ± 0.01 *SD*, *n* = 9), indicating that our image raw data resolve and separate both synaptic markers, as expected.

**Figure 4:**
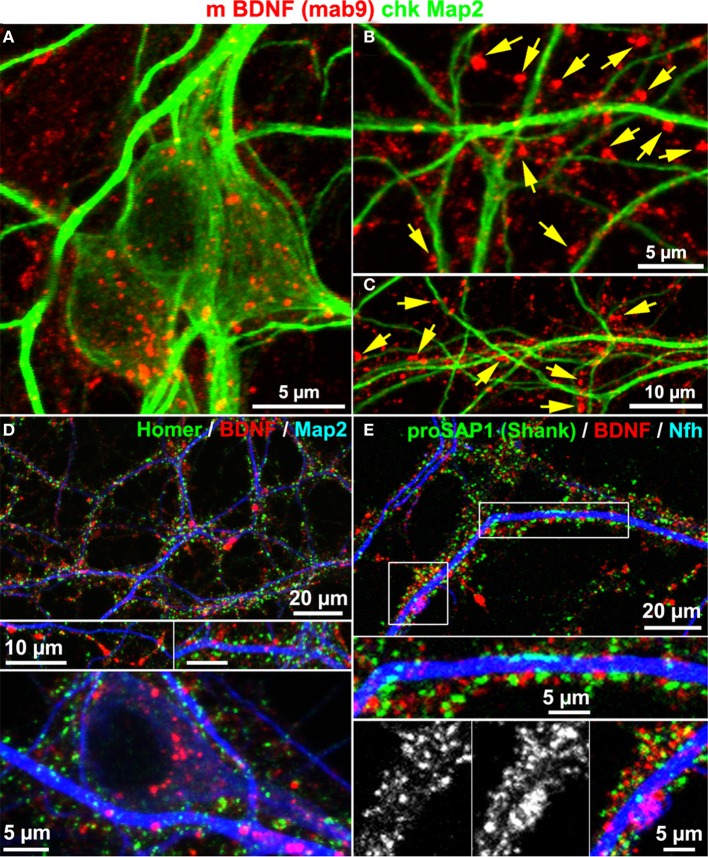
**BDNF immunoreactivity is low in Map2+ neurites. (A)** Map2 and BDNF IR overlapped at somata. **(B–C)** Peripheral synapse-like BDNF labels (yellow arrows) were Map2-negative (DIV 22). **(D,E)** In single confocal image planes, the postsynaptic scaffold proteins Homer1 **(D**; DIV 22**)** and proSAP1 **(E**; DIV 32**)** barely overlapped with BDNF stainings.

Map2 is known to label the somatodendritic contour of neurons, but not dendritic spines or postsynaptic densities (Bernhardt and Matus, 1984). To test whether or not BDNF accumulates in dendrites, we co-stained neurons with anti-BDNF and anti-Map2 antibodies. BDNF IR was rather concentrated along Map2+ dendrites, and was scarcely found to counterstain the Map2 IR. Anti-Map2 stained somatic regions and this signal surrounded the somatic and soma-near BDNF IR (Figure 4A). In peripheral neuritic regions, most BDNF IR clusters were not associated with Map2+ dendrites (Figures 4B,C, yellow arrows), indicating high abundance of BDNF in Map2-negative structures of the neuron. Furthermore, we performed a triple-labeling of BDNF, neurite markers (Map2 or Neurofilament heavy chain, Nfh), and the postsynaptic marker proteins Homer or Proline-rich synapse-associated protein-1 (proSAP1/Shank). Both Homer and ProSAP form a substantial proportion of the postsynaptic density (PSD) core scaffold structure (Sugiyama et al., 2005). The labels for Homer and ProSAP (Shank) aligned along both neurite markers, as generally observed in long-term cultured hippocampal neurons. Interestingly, BDNF IR showed only little overlap with Homer or ProSAP (Shank) signals (Figures 4D,E). Map2 IR showed its typical fibril-like lining of the inner part of dendrites, and no obvious branching into synaptic IR labels.

These results point to synaptic BDNF granules within glutamatergic terminals, but confocal laser scanning microscopy was not able to provide the required spatial resolution to obtain ultimate information whether BDNF is indeed concentrated within presynaptic glutamatergic terminals.

For this purpose, we established super resolution fluorescence imaging of indirect BDNF immunoreactive labels by two-color *direct* stochastic optical reconstruction microscopy (*d*STORM) (Heilemann et al., 2008; Van De Linde et al., 2011).

### *d*STORM imaging of the synaptic scaffold structure

**Figure 5:**
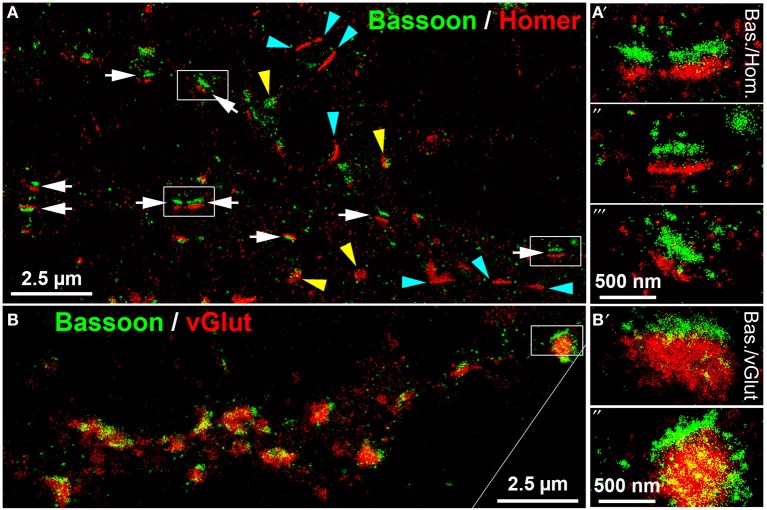
***d*STORM images of Bassoon, Homer and vGluT reveal hallmarks of mature synaptic structures in long-term cultured hippocampal neurons. (A)** Presynaptic Bassoon and postsynaptic Homer1 clusters formed well-separated juxtaposed synaptic bar structures (white arrows) in mouse hippocampal neurons (DIV 35). Elliptical disk-like clusters of Homer1 and Bassoon may represent a “face orientation” of synapses (yellow arrowheads). Some Homer+ bars lacked a presynaptic Bassoon+ counterpart (cyan arrowheads). **(A′^−″′^)** (details), Dense pattern of localization points finally forming a bar-like synaptic scaffold structure. **(B,B′^−″^)** (details), Presynaptic Bassoon bar structures and vGlut+ disks formed an ensemble in which vGlut labels were in tight association with the Bassoon+ scaffold bars. Bas, Bassoon; Hom, Homer.

*d*STORM uses standard fluorescent probes as immunocytochemical tags and switches organic fluorophores between a bright fluorescent on-state and a non-fluorescent off-state (Heilemann et al., 2008; Van De Linde et al., 2011). The determination of the precise position of fluorophores is achieved by approximation of a two-dimensional Gaussian function to the fluorescence emission pattern of individual spatially separated fluorophores in each image frame. *d*STORM images are finally reconstructed from tens of thousands of images, here up to 40,000 image frames per label, that were acquired in an internal reflection fluorescence (TIRF)-microscopy mode. Initially, we performed *d*STORM imaging to resolve the synaptic scaffold proteins Bassoon and Homer. *d*STORM visualized both proteins as dense molecular structures, best described as juxtaposed bars, separated by a synaptic cleft (Figure 5A, white arrows). The trans-synaptic organization of the two synaptic scaffold proteins is pointed out in detail in Figures 5A'–A”'. This synaptic fine structure closely resembles a typical side-view of presynaptic Bassoon and postsynaptic Homer, as described also by Dani et al. (2010) in 3D-STORM images of slices from the glomerular layer of the olfactory bulb (Dani et al., 2010). As expected, presynaptic Bassoon+ bars are in close association with glutamatergic labels on one side of the vGlut+ disc (Figure 5B, and the inset **B',B”**). These data reveal mature synaptic scaffold hallmarks in long-term cultured neurons. However, many Homer+ synaptic bars have no juxtaposed presynaptic Bassoon labels, indicating a mature postsynaptic scaffold, but no presynaptic counterpart (cyan arrowheads in Figure 5A).

### Quantitative analysis of BDNF distribution at synapses

*d*STORM enables single-molecule sensitive super-resolution imaging with a precise separation of different fluorophores, thus being appropriate for co-localization studies of the low-abundant protein BDNF in one color, with high-abundant pre- and postsynaptic markers in a second color. After an initial screening procedure, we found Alexa532 for BDNF labeling and Alexa647 for synaptic markers to be a potent combination for the two-color *d*STORM analysis.

**Figure 6:**
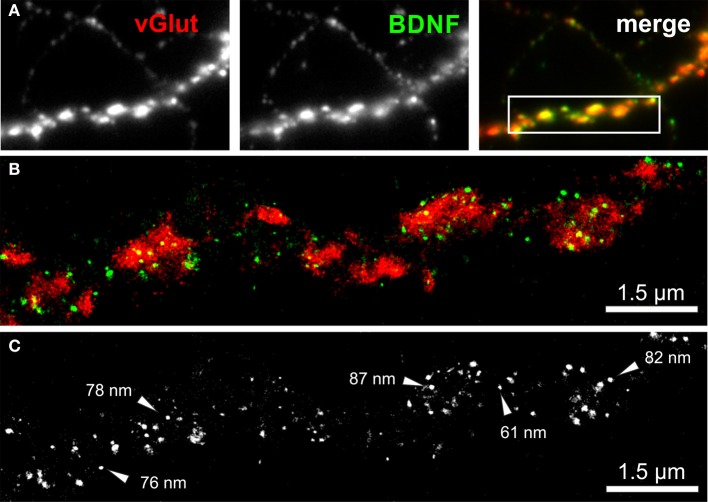
***d*STORM image of BDNF granules within presynaptic glutamatergic terminals. (A)** Epifluorescence image of a peripheral neurite of a hippocampal neuron (DIV 35) with strong labeling of BDNF (green) and vGlut (red). The inset in the overlay image (merge) represents the *d*STORM image shown in **(B)**. **(B,C)** Single BDNF+ granules with a mean diameter of 64 nm were preferentially located within the vGlut+ area, representing the glutamatergic presynapse. Almost 90% of the BDNF immunoreactivity is found to overlap with vGlut. **(C)** Black-white presentation of the BDNF granules from image **(B)**. Single vesicles with a dense BDNF label are pointed out by arrows. These granules have a diameter in the range of 60–90 nm.

**Figure 7:**
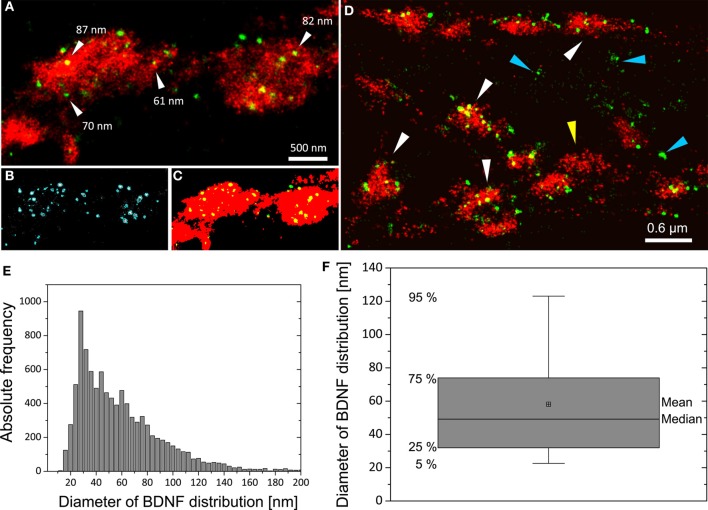
**Distribution pattern of BDNF+ immunoreactivity in *d*STORM images and quantitative analysis of BDNF granules. (A)** In vGlut+ structures BDNF localizations appear as well-separated spherical structures. **(B)** Definition of the BDNF+ distribution within localizations masks (cyan mask). **(C)** Transformation of grayscale image information to a binary information (red vGlut+, green BDNF, yellow color represents the overlap of both labels). Here, 91% of all BDNF localizations overlap with the vGlut+ mask. **(D)** vGlut+ synapses containing densely packed BDNF+ vesicles (white arrows) and vGlut+ discs with low amount of BDNF+ vesicles (yellow arrowhead) were found in close neighborhood. Accumulation of BDNF+ vesicles was also seen at vGlut-negative sites of unknown origin (cyan arrowhead) (here DIV 30). In this example, 67% of the BDNF locations match the vGlut+ label. **(E)** Histogram of the diameter of all BDNF localization points. The selection mask **(B**, cyan mask lines**)** defines the size (pixelarea) of BDNF vesicles. Assuming that the vesicles are almost circular yields to a mean diameter of 58 nm and a median diameter of 49 nm (*n* = 9562 BDNF localizations clusters). **(F)** Boxplot diagram of **(E)**, box defines 1st and 3rd quartile and the whiskers the 5th and 95th percentile.

We imaged either single vGlut+/BDNF+ neuritic structures with *en passant* synapses, or dense peripheral neuritic nets (Figures 6, 7). For a quantitative evaluation of the images, we established binary information masks (see Figures 7B,C) and estimated the percentage of overlapping areas for BDNF and vGlut. In addition, we determined the number and diameter of BDNF granules consisting of 2 or more localization points (see section Materials and Methods). In Figure 6A, a conventional fluorescence image of a vGlut+/BDNF+ neurite is shown. The corresponding *d*STORM images exhibit superior resolution, are almost background-free, and visualize BDNF in small granules within presynaptic vGlut+ synapses (Figures 6B,C). Here, the single neurite with glutamatergic synapses carried 144 BDNF clusters with an average diameter of 64 nm (±3.2 s.e.m.). In this example, the overlap of BDNF clusters with the vGlut counterstain is 88.4%. Remarkable were single spherical presynaptic structures with a diameter of 60–90 nm, which were densely packed with BDNF (Figure 6C; see Figure 7 as well). vGlut+ disc-like synaptic structures are formed by multiple, small spherical localization clusters. This distribution pattern was seen in dense neuritic networks, as well. Here, BDNF clusters were also localized within, or very close to vGlut+ structures, at a distance of only a few nanometers (Figure 7D, white arrows; 150 BDNF IR clusters in this image frame). The overlap with vGlut represents 67.5%. Exemplary vGlut negative/BDNF+ clusters are marked with cyan arrowheads. Some vGlut+ discs with low numbers of BDNF+ localizations exist in close neighborhood to synapses with high BDNF IR (Figure 7D, yellow arrowhead). On average, 39.4% of all BDNF localizations in all x.y-projected *d*STORM images of neurites overlap with immunoreactivity masks of vGlut (% overlap of 12 *d*STORM images; collected from 3 cultures). This value represents all single BDNF IR signals that were collected by *d*STORM imaging. The mean diameter of all BDNF clusters was 58.2 nm (±0.4 s.e.m.; *n* = 9562 clusters, analyzed from 17 *d*STORM images of 4 cultures) (Figures 7E,F).

**Figure 8:**
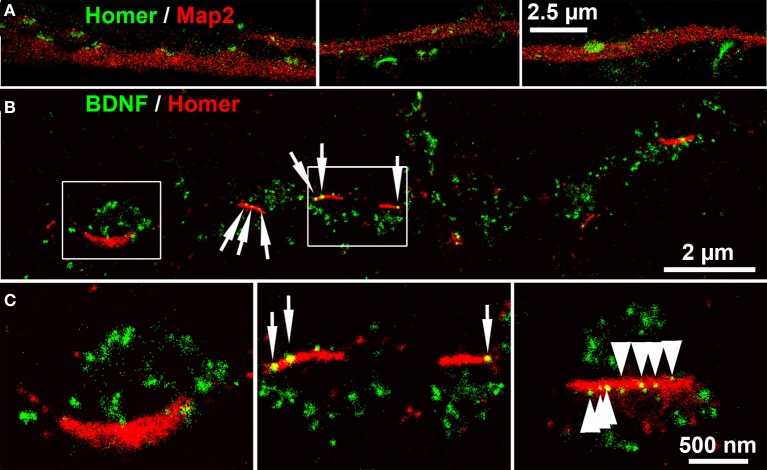
***d*STORM images of BDNF localization at postsynaptic bar structures (DIV 30). (A)** Homer+ bar structures are assembled along, but not within Map2+ dendrites. **(B)** BDNF+ vesicles accumulate in juxtaposed position to Homer+ postsynaptic bars, while only a low number of BDNF+ localizations (6.7%) were found at postsynaptic bar structures (white arrows). **(C) (**left panel, detail of **B)** The inner bow of the Homer+ bar is juxtaposed to BDNF+ clusters. **(**middle panel, detail of **B)**. Some BDNF+ granules overlap with the postsynaptic bars. (right panel) Multiple (nine) small BDNF+ vesicles are aligned within a postsynaptic, Homer+ bar, (arrows) while the vast majority of BDNF localization points do not overlap with the Homer+ scaffold. **(B,C)**: DIV30.

Next we acquired *d*STORM images of BDNF labels of Homer+ postsynaptic bar structures. Homer showed a close association with Map2+ dendrites. Some Homer+ bars were discretely separated from Map2+ dendrites (Figure 8A) (see also the confocal image in Figure 4). At single neurites with *en passant* synapses, the most BDNF+ granules were juxtaposed to Homer+ PSD scaffolds (Figures 8B,C). Only in a few cases, single BDNF signals were also present at these postsynaptic bars (7% of all locations in 5 *d*STORM images, example in Figure 8C).

To conclude, our findings show that long-term cultured hippocampal neurons are able to form synaptic structures with defined attributes of maturity, such as a bar-like organization of synaptic scaffold proteins. At these synapses the vast majority of synaptic BDNF is located within the glutamatergic presynapse.

## Discussion

Here, we performed a super-resolution analysis of endogenous BDNF at glutamatergic synapses of cultured hippocampal neurons. Cultured hippocampal or cortical neurons expressing recombinant BDNF or BDNF fusion proteins have become a standard and often used model to analyze BDNF localization, secretion and function (Canossa et al., 2001; Hartmann et al., 2001; Kohara et al., 2001; Gärtner and Staiger, 2002; Egan et al., 2003; Dean et al., 2009; Matsuda et al., 2009; Orefice et al., 2013). These studies gave insights not only into BDNF biology, but also offered a better understanding of protein secretion in neurons in general. However, for BDNF localization studies in particular, it remained controversially discussed whether natural BDNF is preferentially enriched in the presynapse, in the postsynapse or abundantly present on both sides of the synapse. There are some caveats that need to be considered when BDNF localization is investigated after recombinant expression. First, trafficking of endogenous BDNF has barely been investigated, especially in young neurons, when natural BDNF expression is very low (Maisonpierre et al., 1990). Therefore one cannot judge whether or not BDNF processing and trafficking is well mimicked by overexpressed BDNF. Second, vector-driven BDNF expression can only reflect some of the temporal and spatial aspects of BDNF transcription and splicing when BDNF is expressed from its endogenous promoter sites. A third aspect is that trafficking pathways can be saturated with high amounts of cargo proteins (Blum et al., 2000), a problem which is normally circumvented by an efficient protein sorting machinery (Lippincott-Schwartz et al., 2000). As a consequence, substantial amounts of BDNF might not properly be exported at ER exit sites and would then accumulate in the lumen of the dendritic endoplasmic reticulum.

Here, we used two-color super-resolution fluorescence imaging to localize endogenous, but not overexpressed BDNF at synapses. The synaptic network of neurons in a hippocampal culture is built up by neurons with hundreds of synapses along the shafts of their axons and axon collaterals. These synapses are best described as *en passant* synapses and our data show a high abundance of BDNF-containing granules directly within presynaptic glutamatergic terminals. A minor amount of BDNF immunoreactivity was present at postsynaptic bars opposite BDNF-rich presynaptic regions, as well.

*In vivo* BDNF is also present in *en passant* projections connecting the enthorinal cortex layer III with the stratum lacunosum-moleculare, at presynapses acting at apical dendrites of the hippocampal CA1 pyramidal neurons (Dieni et al., 2012). In CA1, BDNF immunogold labeling and electron microscopy revealed pleiomorphic, vesicle-like clusters directly in axonal endings in the stratum radiatum of the CA1 region. In this region most excitatory synapses are glutamatergic and formed by the Schaffer collateral or by projections from the enthorinal cortex (Dieni et al., 2012). BDNF is also stored in discrete vesicles within the mossy fiber boutons (MFB) and some MFB-filaments (Danzer and McNamara, 2004; Dieni et al., 2012). In the glutamatergic MFBs, BDNF-containing clusters do colocalize neither with the synaptic vesicle markers synaptophysin or vGlut1, nor with proteins that are stored in dense core vesicle fractions. All these findings support the view of a preferential location of BDNF in glutamatergic presynapses, as cargo of specific dense core vesicles (Dieni et al., 2012). However the biochemical composition of the BDNF-containing vesicle remains a puzzling issue. We describe BDNF immunoreactive clusters consisting of multiple localizations in discrete synaptic granules with a size from 25 to 140 nm. These granules are not organized along presynaptic bar structures, but were found to be distributed over the whole presynapse. This may have consequences for the BDNF release mechanism and it will be a challenging issue to find out whether or not BDNF is released at the active zone of the synapse.

On the postsynaptic site, some rare BDNF-containing clusters aligned along the postsynaptic bar (Figure 8C; right panel). Particularly, we obtained no evidence of BDNF IR in a tubule-like or network-like pattern within the smooth ER in dendrites. Therefore we prefer the interpretation that these vesicles represent endosomes that are transcellularly filled from the BDNF-rich presynaptic part, probably via the endocytosis together with postsynaptic TrkB receptors (Kohara et al., 2001; Lazo et al., 2013; Orefice et al., 2013). A recent study argues against the local formation of BDNF in dendrites. Deep sequencing and high-resolution localization confirmed that most of the BDNF transcripts are present in the somatic compartment of hippocampal slices or localize to the somata of cultured hippocampal neurons (Will et al., 2013). BDNF transcripts were only rarely detected in the dendritic processes (Will et al., 2013). In context with our study here these data suggest that BDNF protein is most likely formed in the somatic compartment and brought to the glutamatergic presynapse by anterograde transport.

It has been discussed that BDNF-containing endosomes might form an additional source of BDNF at synapses (Luikart and Parada, 2006; Park and Poo, 2012). A speculative assumption is that at mature synapses most BDNF is released from the presynapse, taken up by the postsynapse and is re-offered to the presynapse to modulate presynaptic transmitter release. It has been shown that BDNF facilitates transmitter release from presynapses via the phosphorylation of synapsins (Jovanovic et al., 2000). Given that BDNF has a very high affinity to its receptor TrkB, one can assume that even single BDNF molecules may be sufficient to cause a fast and local action (Blum and Konnerth, 2005). At present, it is technically impossible to visualize the secretion of endogenous BDNF at single synapses, but such a technique would allow the monitoring of regulated synaptic BDNF secretion in contrast to the classical global BDNF secretion assays.

In our cultured hippocampal neurons, we found no evidence for BDNF within GABAergic synapses. However, it is important to note that we cannot provide ultimate proof that synaptic BDNF is exclusively present in the glutamatergic terminal. To provide conclusive data about the absence of BDNF in GABAergic synapses genetic tools will be needed that enable to distinguish between the cell-type specific production of BDNF and local uptake of BDNF via receptor-mediated endocytosis, e.g., via TrkB or TrkC.

Despite the caveat of exogenous expression of BDNF in these studies, it has been confirmed that quite young cultured neurons develop an efficient machinery to secrete BDNF from dendrites (microcultures at DIV 9–11) (Brigadski et al., 2005). Even backpropagating action potentials provide sufficient dendritic excitation to trigger local, dendritic secretion of BDNF at DIV 13–14 (Kuczewski et al., 2008). The release of a pH-sensitive fluorescent protein-tagged BDNF from dendrites at DIV 14–21 required lower levels of neuronal spiking than axonal release (Matsuda et al., 2009), an effect that might point to differences in the source of calcium to induce calcium-dependent neurotrophin release (Blum and Konnerth, 2005). Regarding the hippocampus, our knowledge of the temporal pattern of synaptic maturation and a potential role of dendritic protein secretion is not known. It will be an interesting challenge to unravel the developmental biology of dendritic vs. axonal aspects during synapse refinement *in vivo*. In the near future, subdiffraction-resolution fluorescence imaging will help us shed more light on the maturation process of synapses, and the developmental process underlying the formation of a functional neurotrophin release site. In particular, single-molecule localization microscopy methods as used here are ideal tools to extract quantitative data about the molecular organization of pre- and postsynaptic sites.

## Author contributions

Markus Sauer, Robert Blum conceived and designed research; Thomas Andreska, Sarah Aufmkolk, Robert Blum performed research; Thomas Andreska, Sarah Aufmkolk, Robert Blum analyzed data; Sarah Aufmkolk, Markus Sauer, Robert Blum wrote the paper.

### Conflict of interest statement

The authors declare that the research was conducted in the absence of any commercial or financial relationships that could be construed as a potential conflict of interest.
